# Small Interfering RNAs Are Highly Effective Inhibitors of Crimean-Congo Hemorrhagic Fever Virus Replication In Vitro

**DOI:** 10.3390/molecules25235771

**Published:** 2020-12-07

**Authors:** Fanni Földes, Mónika Madai, Henrietta Papp, Gábor Kemenesi, Brigitta Zana, Lili Geiger, Katalin Gombos, Balázs Somogyi, Ildikó Bock-Marquette, Ferenc Jakab

**Affiliations:** 1 National Laboratory of Virology, BSL-4 Laboratory, Szentágothai Research Centre, University of Pécs, 7624 Pécs, Hungary; fanni4444@gmail.com (F.F.); mmoni84@gmail.com (M.M.); phencsi@gmail.com (H.P.); kemenesi.gabor@gmail.com (G.K.); brigitta.zana@gmail.com (B.Z.); sobalbundi@gmail.com (B.S.); 2 Institute of Biology, Faculty of Sciences, University of Pécs, 7622 Pécs, Hungary; 3 Department of Laboratory Medicine, Medical School, University of Pécs, 7624 Pécs, Hungary; g.lilly92@gmail.com (L.G.); gombos.katalin@pte.hu (K.G.); 4 Regenerative Science, Sport and Medicina Research Group, Szentágothai Research Centre, University of Pécs, 7624 Pécs, Hungary; ibockm@gmail.com

**Keywords:** CCHFV, Nairovirus, siRNA, RNA interference, gene silencing

## Abstract

Crimean-Congo hemorrhagic fever virus (CCHFV) is one of the prioritized diseases of the World Health Organization, considering its potential to create a public health emergency and, more importantly, the absence of efficacious drugs and/or vaccines for treatment. The highly pathogenic characteristic of CCHFV restricts research to BSL-4 laboratories, which complicates effective research and developmental strategies. In consideration of antiviral therapies, RNA interference can be used to suppress viral replication by targeting viral genes. RNA interference uses small interfering RNAs (siRNAs) to silence genes. The aim of our study was to design and test siRNAs in vitro that inhibit CCHFV replication and can serve as a basis for further antiviral therapies. A549 cells were infected with CCHFV after transfection with the siRNAs. Following 72 h, nucleic acid from the supernatant was extracted for RT Droplet Digital PCR analysis. Among the investigated siRNAs we identified effective candidates against all three segments of the CCHF genome. Consequently, blocking any segment of CCHFV leads to changes in the virus copy number that indicates an antiviral effect of the siRNAs. In summary, we demonstrated the ability of specific siRNAs to inhibit CCHFV replication in vitro. This promising result can be integrated into future anti-CCHFV therapy developments.

## 1. Introduction

Crimean-Congo hemorrhagic fever virus (CCHFV) categorically belongs to the *Orthonairovirus* genus, the *Nairoviridae* family in the *Bunyavirales* order. CCHFV is causing a mild to severe hemorrhagic disease in humans, with fatality rates from 5% up to 30% [1].

CCHFV is characterized by a tripartite single-stranded RNA genome (S, M, and L segment) of ambisense (S) and negative (M, L) polarity. The three genome segments encode four structural proteins: the RNA dependent RNA polymerase is encoded by the large (L) segment, the glycoproteins (G_N_ and G_C_) are encoded by the medium (M) segment, and the nucleocapsid protein and nonstructural protein are encoded by the small (S) segment [2].

Emerging infectious diseases (EIDs) are growing threats to animal and human health throughout the world. CCHFV is a tick-borne pathogen that causes an increasing number of severe infections and presents over a wide geographic range, including areas in South-Eastern Europe, Western and Central Asia, the Middle East, and Africa as well [1]. This virus is transmitted primarily by ticks, but the spectrum of natural hosts for CCHFV includes a wide variety of domestic and wild animals [3].

There are neither vaccines nor effective antiviral therapies for the treatment of CCHFV infections in humans to date [4]. There is a growing need for advanced research and development activities for such pathogens as CCHFV, since there is a constantly growing geographic and epidemiologic burden of the disease and BSL-4 capacity is limited throughout the world, which can safely handle such research.

Among antiviral therapies, RNA interference (RNAi) can be used to suppress viral replication by targeting either viral- or host genes that are needed for viral replication. Since its discovery in 1998 [5], it has revolutionized the mechanism of gene silencing and improved our understanding of the endogenous mechanism of gene regulation to enhance the use of new tools for antiviral research. Silencing viral genes such as viral polymerases, master regulators of viral gene transcription, and viral genes that act early in the viral life cycle may suppress viral replication more effectively than targeting late or accessory viral genes. Moreover, RNAi could target viral proteins and pathways, which are unique to the viral life cycle, and it has become possible to interfere with viral infections and replication without unacceptable host cell toxicity [6]. Accordingly, the major advantage of RNA interference is its target specificity. In recent years, many viruses have been successfully targeted by RNA interference such as human immunodeficiency virus (HIV) [7,8], Severe Acute Respiratory Syndrome coronavirus (SARS-CoV) [9], Hepatitis B virus (HBV) [10], Hepatitis C virus (HCV) [11], Influenza A virus [12], Hazara virus (HAZV) [13], Langat virus (LGTV) [14], Andes virus (ANDV) [15] and West Nile virus (WNV) [16]. So far, to the best of our knowledge, this is the first study that used RNA interference to inhibit CCHFV replication in vitro. Although, gene silencing by RNA interference was published in association with another member of the *Nairoviridae* family, the Hazara virus [13].

RNA interference uses small double-stranded RNAs with a complementary sequence to the target silencing genes. Nevertheless, endogenous gene silencing operates through multiple mechanisms such as mRNA cleavage, inhibition of translation, and epigenetic modifications of chromatin, of which mRNA cleavage is the most efficient mechanism for antiviral therapies [6]. Small interfering RNAs (siRNA) are the active agents in RNA interference. The siRNAs are 21–22 nucleotides long, serve as a guide for cognate mRNA degradation [17]. Naturally, these siRNAs are a result of endonucleolytic processing of a larger precursor RNA. Experimentally, RNAi can be triggered in mammalian cells after the transfection of synthetic siRNA using suitable transfection reagents. These siRNAs are incorporated into a cytoplasmic RNA-induced silencing complex (RISC), which cleaves exogenous double-strand siRNAs, leaving an unpaired guide strand to search for complementary mRNAs. If the target site on the mRNA has nearly perfect complementarity to the guide siRNA, the mRNA is cut by an Argonaute (Ago) endonuclease in the RISC and is degraded. This way, siRNA is silencing the expression of the protein encoded by the target mRNA. Typically, protein expression is reduced but not eliminated [6].

Recent works have shown that more effective antiviral therapies are urgently needed to treat virus infections especially for viruses with growing epidemic potential [9,15]. Furthermore, these RNA interference experiments have shown promising results in vivo [18,19], and there are many limits to their application in vivo. Many aspects of the CCHFV cell entry, replication, and pathogenesis remain poorly defined. It was mostly studied by using minigenome systems or virus-like-particle systems considering its highly infectious nature and the lack of BSL-4 laboratories [2]. In our present study, we aimed to design chemically synthesized siRNAs that can inhibit CCHFV replication in vitro. This study presents the first step forward to future RNAi-based CCHF antiviral therapy development. Although RNA interference is an ancient cellular antiviral response and a long-standing research topic, in recent years, there has been a large increase in experiments for its use against diseases. The novelty of our study originates from the fact that RNAi has not yet been applied in the case of CCHFV.

## 2. Results

In our study, nine siRNAs were designed and synthesized to test the inhibitory activity in vitro on CCHFV replication and target the mRNAs produced by S, M, and L segments. We detected the high inhibitory effect of S (siS2), M (siM1), and L (siL3, siL4) segment-specific siRNAs. We experienced that siRNAs inhibited CCHFV replication in different efficiencies and a dose-dependent manner.

### 2.1. Cytotoxicity Tests

During the experiments, two different types of cell viability tests were used: light microscopic observation and luminescence cytotoxicity measurement.

The siRNAs treatment could cause visual cytopathogenic effects (CPEs) and affect viral growth, therefore we performed light microscopic observation to evaluate cell growth and viability. Firstly, we had to find the appropriate siRNA concentration that is effective in inhibiting CCHFV replication but not toxic to the cells. In our cytotoxicity experiments, after three days of siRNAs transfection, the cell number per well was observed and compared to non-transfected cells by manual counting with a hematocytometer. In these experiments, we did not detect the cytotoxic effect of siRNAs on A549 cells at any lower concentrations used. However, a high concentration of siRNAs caused cell morphology changes and cell death.

**Figure 1:**
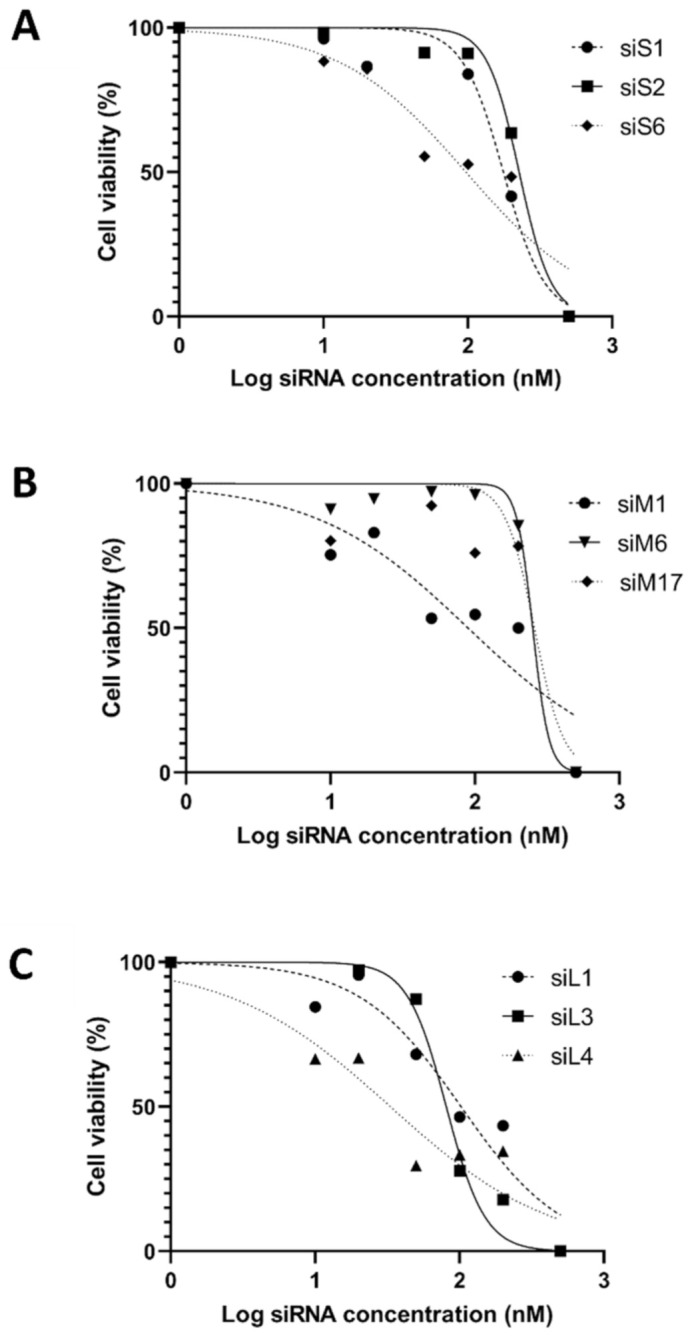
The graphs show cell viability assay results by GraphPadPrism software. (**A**) Represents the effect of different concentrations of S segment siRNAs, (**B**) M segment siRNAs, and (**C**) the L segment siRNAs. Calculated CC50 and R squared data are represented in Table 1. The horizontal axis represents the logarithmized, different concentrations of siRNAs (nM) and the vertical axis represents the cell viability (%).

In addition to morphological observation with light microscopy, luminescence cytotoxicity measurements (Promega–Cell Titer Glo Luminescent assay) were used. The cytotoxic concentration of the extracts caused death to 50% (CC50%) of viable cells in the host. The CC50 was calculated using GraphPadPrism version 8.00 software (Graph Pad Software, San Diego, CA, USA) for non-linear regression (Figure 1). In most cases, during cell viability tests, results that were observed microscopically were the same as the luminescence cytotoxicity measurements. Compared to the cell control and transfection reagent control (Lipofectamine RNAiMax transfection reagent was added to A549 cells) mean cytotoxicity values gave almost identical values and showed no cell cytotoxicity in the luminescence cytotoxicity measurements.

In the case of siRNAs against the CCHFV S segment, the results were the same with the cell viability tests: CC50 was observed around 100 to 200 nM in every siRNAs against the S segment (CC50_siS1_ = 177.7 nM; CC50_siS2_ = 246.7 nM, CC50_siS6_ = 106.5 nM). The highest S segment siRNA (siS2) CC50 value was 246.7 nM, which was calculated by GrapPhadPrism version 8.00 software. CCHFV M segment siRNAs were proven to be non-toxic for the A549 cells around 100 to 300 nM concentration (CC50_siM1_ = 99.84 nM; CC50_siM6_ = 316.8 nM, CC50_siM17_ = 298.8 nM). The highest M segment siRNA (siM6) CC50 value was extremely high (316.8 nM) compared to other siRNAs. In the case of siRNAs against the CCHFV L segment, results were the same as the cell viability tests. The L segment siRNAs (siL3, siL4) CC50 values were 80.92 and 54.29 nM, respectively. SiL1 siRNA CC50 value was 109.7 nM. However, siRNAs against all of the segments were used in a maximum of 50 nM concentration because of the comparability. The highest concentration that was used during experiences determined by the lowest CC50 value of siRNAs (Table 1).

Summarizing the cell viability results, the minimum concentration of siRNAs was set to 10 nM, and the maximum concentration to 50 nM in the case of every segment during the experiments. The little differences found between the cytotoxicity tests indicate that the use of microscopic observation alone is not sufficient enough to detect cell viability and specify the appropriate concentration of siRNAs.

### 2.2. Inhibition of CCHFV Replication Using Segment-Specific siRNAs

**Figure 2:**
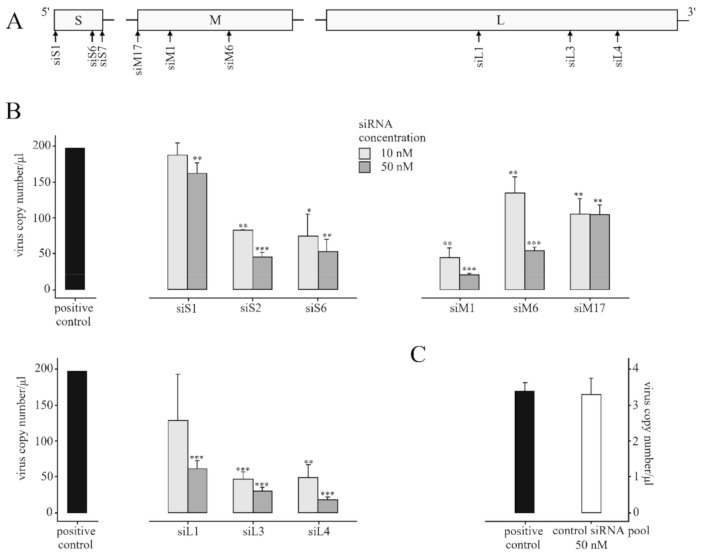
A549 cells were transfected with siRNAs, which were designed for Crimean-Congo hemorrhagic fever virus (CCHFV) S, M, and L segments in different concentrations (10 nM, 50 nM). After transfection, cells were infected with CCHFV at a MOI of 0.1. Three biological replicates of siRNA inhibited CCHFV samples were used and the positive control was used in three biological and three technical repeats. The virus copy number was determined after 72 h by RT-ddPCR. (**A**) CCHFV schematic gene map containing the designed CCHFV-specific siRNAs site; (**B**) inhibitory effect of siRNAs against CCHFV: the horizontal axis represents the virus copy number/µL and the vertical axis represents the positive control and designed siRNAs. Student’s *t*-tests were significant if *: *p* < 0.05, **: *p* < 0.01, ***: *p* < 0.001. Error bars represent the standard deviation (SD) of the means for three independent experiments. (**C**) Positive control and ON-TARGET plus non-target siRNA pool comparison: there is no significant difference between them by Student’s *t*-tests. The horizontal axis represents the virus copy number/µL and the vertical axis represents the positive control and non-targeting control siRNA pool. Error bars represent the standard deviation (SD) of the means for three independent experiments.

In our experiments, nine siRNA were designed against all segments of CCHFV (Figure 2A). Based on the RT-ddPCR results, a high and significant copy number decrease was detected in the case of some siRNAs (siS2, siM1, siL3, and siL4).

As shown in Figure 2B, when siS2 was used at 50 nM concentration, it strongly and significantly inhibited CCHFV replication compared to the positive control (*p* < 0.001). Among siRNAs against CCHFV S segment, siS2 was the most efficient inhibitory siRNA. Furthermore, siS6 has shown a moderate but significant inhibitory effect in CCHFV replication (*p* < 0.01). In contrast, the significant antiviral inhibitory effect of siS1 at 10 nM concentration was not detected.

Between siRNAs, which were designed for the M segment, siM1 had strong and significant antiviral activity at 50 nM concentration (*p* < 0.001). Moreover, siM6 has also shown CCHFV inhibitory effect at the medium level (*p* < 0.001) at 10 nM concentration but strong and significant antiviral activity at 50 nM concentration (*p* < 0.001). In contrast, siM17 did not inhibit CCHFV replication significantly at neither 10 or 50 nM concentrations.

In the case of the L segment, when siL4 was used at 50 nM concentration, it strongly inhibited CCHFV replication compared to the positive control (*p* < 0.001). SiL3 has also shown significant, high activities on CCHFV replication at 50 nM concentration, while siL1 has shown nearly the same efficiency at 50 nM concentration (Figure 2B).

We compared the amount of inhibitory effect of different siRNAs to the positive control. We did not use ON-TARGET plus non-targeting siRNA pool as a benchmark because it gave a result very similar to the positive control. Thus the positive control is suitable for comparison with the inhibitory effect of the siRNAs (Figure 2C). The difference in copy number scale between experiments is given by the separate experiments and the degrees of dilution.

At least one highly inhibitory siRNA was found in the case of every segment. The siRNAs that were designed for the S segment: the siS2 has shown an efficient decrease in the virus copy number by about 77% at 50 nM concentration. In the case of M segments siRNAs, siM1 has decreased the virus copy number by about 90% at 50 nM concentration. Among siRNAs that were designed for the L segment, the most effectively siL4 affected CCHFV replication (decrease by about 91%) at 50 nM concentration just like siM1. The inhibitory effect against CCHFV was not caused by siS1 and siM17.

**Figure 3:**
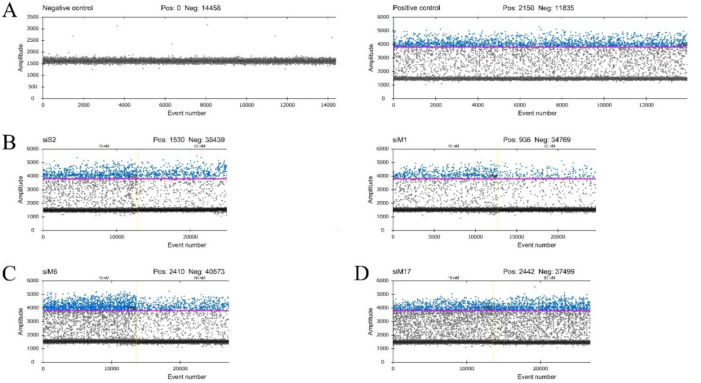
QuantaSofts’ RT-ddPCR fluorescent readouts. The horizontal axis represents the event number and the vertical axis represents the fluorescence amplitude in the FAM channel. The strict threshold line (pink line) was set for every sample at 3780 amplitude. Positive droplets were represented in blue and negative droplets were represented in grey. (**A**) The negative control sample was shown as the negative droplet population without positive droplets, the positive control sample was shown as the extensive positive droplet population; (**B**) siS2 and siM1, respectively, showed a concentration-dependent high inhibitory effect, different concentrations (10 nM, 50 nM) were separated with a yellow, dotted line; (**C**) siM6 showed a concentration-dependent medium inhibitory effect at different concentrations (10 nM, 50 nM) that were separated with a yellow, dotted line; (**D**) siM17 has shown low inhibitory effect against CCHFV at different concentrations (10 nM, 50 nM) that were separated with a yellow, dotted line.

QuantaSofts’ RT-ddPCR raw fluorescence readouts have shown negative and positive controls in Figure 3. A negative droplet population was shown by the negative control sample without any positive droplets. The positive control sample has appeared as a massive positive droplet population above the threshold level. In the case of the positive control sample, the positive droplet “rain” was caused by the high concentration of CCHFV and appeared as a background signal. The concentration-dependent high inhibitory effect was shown by siS2 and siM1. At 10 nM concentration, the positive droplet number was high in the case of siS2, however, at 50 nM concentration, the positive droplet number was decreased. SiM1 acted similarly to siS2. A medium inhibitory effect against CCHFV replication was presented by SiM6. The positive droplet number decreased moderately from 10 to 50 nM compared to siS2 and siM1 events. In the case of siM17, the significant inhibitory effect was not detected.

## 3. Discussion

Therapeutic options for the treatment of CCHFV infection are lacking, with the noticeable exception of ribavirin, which is currently recommended by the WHO. Nevertheless, novel and more sophisticated antiviral therapies against nairovirus infections are urgently needed. In the last few years, several studies have shown that siRNAs have the potential to be operated as a specific therapeutic strategy against some viral infections [11,13,14,20]. However, most of these experiments are in the in vitro test phase, and translating RNAi in the clinic, as a conventional treatment option remains a pivotal challenge. In the case of in vivo therapies, one of the most difficult parts is efficiently and specifically delivering siRNA to target tissues and cells. Moreover, the poor cellular uptake of siRNAs in combination with rapid enzymatic degradation are limiting RNAi usage in vivo therapies. Because of the association of siRNAs with a non-target gene, an off-target effect can occur. The off-target effect is the other main limitation of in vivo applications. Furthermore, different classes of siRNA chemical modifications can increase the efficiency of delivery. Fortunately, despite difficulties in virus entry, cytotoxicity and the stimulation of unspecific immune response researches evolved and reached in vivo experiments [18].

One of the main influencing factors in RNAi experiments is the off-target effect, in which siRNAs do not bind to the target gene, but induce silencing in other non-targeted genes. Caffrey and colleagues studied that the incidence of nonspecific targeting can depend on the concentration of the siRNA, with a higher concentration leading to a greater off-target effect [21]. Therefore, taking into account the results obtained from the cytotoxicity assay, we kept the concentration of siRNAs low, and we maximized it to 50 nM to minimalize off-target effects. Antiviral-response pathways, inducing the expression of antiviral-response genes are stimulated by high concentrations of siRNAs in the cells [22].

We evaluated the antiviral activity of siRNAs targeting the S (nucleoprotein), M (glycoproteins), and L (polymerase) transcripts of CCHFV for the first time in vitro. The siRNAs were designed for each CCHFV segment in an effort to find the most effective ones. We observed that among nine tested siRNAs, almost half of them (siS2, siM1, siL3, siL4) were capable of reducing CCHFV copy numbers by more than about 70% during in vitro infection studies, compared to the positive control. However, strong inhibition of CCHFV replication (by about 90%) was performed by only two siRNAs (siM1, siL4). The CCHFV RNA decrease was observed with RT-ddPCR, which does not completely demonstrate infectious virus particles but the purpose of our experiment was to establish the ratios of CCHFV RNA decrease and effect/non-effect of siRNAs. The unusual ability of many siRNAs to inhibit the virus, contrary to previous studies, is due to the successful design and the high rate of transfection achieved.

In the case of the CCHFV S segment protein, nucleoproteins play a central role in the regulation of viral replication. Nucleoprotein has been associated with genomic viral RNA to form RNPs and provide a template for the polymerase. In the last few years, several homologous interferences have been described as the inhibition of the S segment of other nairoviruses by siRNAs and suppression of viral replication [13,15,20]. Levin et al. found that Akabane virus (AKAV) infected Vero cells indicated more than 99% inhibition [20], whilst Chiang et al. described siRNA against the S segment of Andes virus (ANDV) greatly reduced levels (>60%) of viral protein expression [15]. In the case of the Hazara virus inhibition, the siRNAs, which were designed against the S segment, had a higher effect (up to 90%) than those targeting M and L segments [13]. Several experiments performed has shown that the S segment of the genus *Orthobunyavirus* is the RNA interference prime target in arthropod cells [23,24]. In our study, among siRNAs that were designed against the S segment, siS2 has inhibited the CCHFV copy number effectively. Our study is in agreement with previous works [13,15] claiming that targeting the S segment by siRNAs can produce an effective inhibitory impact. In consequence, using the S segment as the target for silencing virus replication has proven to be an option for future therapeutics. Hereafter, using siRNAs together can have a superior effect against virus infections [20]. Our plans include the combined and pooled use of designed siRNAs against CCHFV infection.

CCHFV glycoproteins (Gn, Gc) are involved in cell entry, initial binding, and fusion. However, the details of specific glycoprotein involvement remain unknown [2]. In contrast with other studies, a high inhibitory effect of siRNA (90%) was found against the M segment. Furthermore, Chiang et al. described that viral glycoproteins are limiting factors for virus production and viral glycoproteins are detected mainly in the lysosome rather than on the cell surface in genus *Orthonairovirus* endothelial cells. In that study, reducing the glycoprotein levels with siRNA against the M segment had a greater impact on virus copy number (decrease by about 90%) and release [15]. Moreover, the M segment is the most diverse genome of CCHFV. This diversity may come from how CCHFV uses the vectors and vertebrate hosts in different geographic ranges. Therefore, it is difficult to design general well-functioning inhibitory siRNAs for this segment and many studies found a lower inhibitory effect. Although glycoproteins encoded by the M gene are the most variable portion of the CCHF viruses, some functional domains of the glycoproteins are well conserved [2,25].

In the case of CCHFV, the largest of the three segments termed the L segment, encodes an RNA-dependent RNA polymerase (RdRp) that is characterized by several conserved functional regions [2]. Moreover, next to nucleoprotein, L protein drives the processes of transcription and replication that occur in the cytoplasm during the viral replication cycle. Thus, targeting this segment is likely to be an exact strategy. We found that siL4 caused 90% reduction in the CCHFV copy number compared to the positive control.

Taken together, our results provide further support for the use of RNA interference-based techniques in the development of antiviral drugs against CCHFV infections. To our knowledge, this is the first study that used designed siRNAs against CCHFV replication in vitro and the first study to provide RNAi solution to all three genomic segments of a nairovirus in parallel. Currently, CCHFV constitutes a notable public health concern in our region, with significant geographic expansion in recent decades and growing epidemic potential [26,27,28]. One major limitation of our study is the lack of combinative experiments; however, it projects future research directions well. Combining efficient siRNAs with each other may reveal their potential synergic inhibition effect. Accordingly, the threat of viral infection will increase in the coming years, so any kind of research project aimed at preventing and overcoming a possible infection may be useful. Moreover, we would like to design time-dependent experiments that examine siRNAs efficiency before and after CCHFV infection. This study gives novel and important research results for one of WHOs prioritized emerging diseases and constitutes a major step for future antiviral development efforts.

## 4. Materials and Methods

### 4.1. Cell Line, Virus Amplification and Titer Determination

A549 cells (human lung carcinoma cell line, ATCC CCL-185) were grown in Dulbecco’s modified eagle medium (DMEM) (Lonza, Basel, Switzerland) supplemented with 10% heat-inactivated fetal bovine serum (FBS) (Euroclone S.p.A,. Pero, Italy) and 1% Penicillin-Streptomycin (Lonza) maintained at 37 °C in a humidified atmosphere containing in a 5% CO_2_.

A549 cells with 60% confluence were infected by the CCHFV Kosova Hoti strain [29] in our experiments. The virus was grown to high titers on A549 cells and the supernatants were aliquoted and were frozen at −80 °C in 1 mL vials and constituted the viral stock. All laboratory manipulations associated with infectious CCHFV were performed in a BSL-4 suite laboratory, aligned to the University of Pécs, Szentágothai Research Centre.

CCHFV viral stock was titrated using the TCID50 method with the immunofluorescence assay. Briefly, serial 10-fold dilutions of CCHFV supernatant were inoculated (100 µL) on 60% confluent A549 cells (30,000 cells/well) in 48-well plates. Viral adsorption was allowed for 1 h at 37 °C. After washing cells with PBS three times, cells were incubated for 3 days at 37 °C in DMEM supplemented with 2% FBS. The fixation and the immunofluorescence assay were performed as previously described using with polyclonal mouse antibody, which was produced against the recombinant CCHFV capsid protein [27]. The percentage of infected cells was observed with immunofluorescence microscopy and recorded for each virus dilution, then results were used to mathematically calculate a TCID50 result with the Spearman-Karber method. During our experiments, A549 cells were infected with CCHFV at an MOI of 0.1 in our following infection and transfection assays.

### 4.2. Design and Synthesis of siRNAs

The sequences of CCHFV Kosova Hoti strain S, M, and L genomic segments (GenBank: DQ133507, EU037902, EU044832) were used to design the siRNAs. Synthetic 21-nucleotide siRNAs with short 3′ overhangs (UU) were designed by the Whitehead siRNA Selection Program to have an antisense strand complementary to the CCHFV [30]. The siRNA sequences were chosen according to the algorithm score. For each viral mRNAs, three siRNAs were synthesized by Dhramacon^TM^ (Table 2). Sequences were subjected to a BLAST search against GenBank to minimize off-target effects. All lyophilized siRNAs were reconstituted according to the manufacturer’s instruction, aliquoted in 10 µM stock solutions, and were stored at −20 °C until further use. The TOX siRNA (siTOX) (Dharmacon^TM^ RNAi technologies, Lafayette, USA) was used to determine transfection efficacy. ON-TARGET plus non-targeting siRNA pool (Dharmacon^TM^ RNAi technologies, Lafayette, USA) was used as control siRNA, which causes minimal changes in treated cells and reflects a baseline cellular response that can be compared to the levels in cells treated with target-specific siRNAs.

### 4.3. Transfection Efficiency

For each experiment, transfection efficiency was monitored by transfecting A549 cells with 200 nM of siTOX (Dharmacon^TM^) under the same experimental conditions. Cells successfully transfected with siTOX went under apoptosis and cell death within 24–48 h. After 3 days of incubation, siTOX transfected cells were trypsinized and manually counted using a hematocytometer (Trypan blue exclusion assay). Transfection efficiency was calculated as the ratio between the numbers of viable siTOX-transfected cells versus non-transfected cells. In our experiments, we experienced an average of 80% transfection efficiency.

### 4.4. Cytotoxicity Tests

In some cases, the designed siRNAs could interfere with the tested cells’ genes (off-target effect) and cause cell death. During the concentration-dependent transfection, the microscopic observation was performed. A549 cells were transfected with different concentrations (ranging from 0.1 nM to 300 nM) of siRNAs. Cells were observed microscopically after the transfection at 24, 48, and 72 h. During the trypan blue exclusion assay, cell deaths and cell morphological changes have been recorded if the siRNAs targeted S, M, or L segments of CCHFV at high siRNA concentration.

Besides microscopic observation, cell cytotoxicity was examined with a luminescence cell viability assay kit (Promega–Cell Titer Glo Luminescent assay). This method determines the number of viable cells in culture, based on quantitation of the ATP present. Cells were transfected with different concentrations of siRNAs (ranging from 0.1 nM to 200 nM). The transfection reagent, Lipofectamine RNAiMax (Thermo Fisher Scientific) cytotoxicity was also tested. The final volume of Lipofectamine RNAiMax was 1.5 µL/well during the luminescent assay. After 72 h of transfection, the luminescence measurement was performed. The CC50 was calculated using GraphPadPrism version 8.00 software (Graph Pad Software, San Diego, CA, USA) for non-linear regression.

The use of cytotoxicity tests was important to find out the concentration at which siRNAs do not cause cell death but their concentration is high enough to inhibit virus replication.

### 4.5. Transfection and Infection Assay

Transfection and infection experiments were performed on A549 cells in the BSL-4 laboratory. A549 cells were seeded in 96-well plates at a density of 2 × 10^4^ cells/well to achieve 60–70% confluent cell monolayers on the day after in a humidified incubator at 37 °C with 5% CO_2_.

Cells were transfected in triplicate biological replicates with siRNAs in the following final concentrations: 10 to 50 nM. Various siRNA concentrations were complexed with the transfection reagent Lipofectamine RNAiMax transfection reagent (Thermo Fisher Scientific, Waltham, MA, USA) according to the manufacturer’s instructions. The transfection reagent and siRNAs were diluted in Opti-MEM medium (Gibco). The final volume of Lipofectamine RNAiMax was 1.5 µL/well. The transfection mixture was incubated for 20 min at RT to allow the formation of siRNA-lipid complexes and 100 µL of the solution was added slowly dropwise to each well. Mock transfected, non-transfected A549 cells were used as controls for the experiments. ON-TARGET plus non-targeting siRNA pool (Dharmacon^TM^ RNAi technologies, Lafayette, CO, USA) was used as a control siRNA. Mock-transfected cells go through the transfection process without the addition of siRNA while non-transfected cells have not been treated at all. At 16 h post-transfection, cells were gently washed twice with DMEM. Thereafter transfected cells were infected with CCHFV at an MOI of 0.1. The inoculum was incubated for 1 h to allow the absorption of the virus on transfected cells. Cells were then cultivated in DMEM supplemented with 2% FBS, 1% Penicillin-Streptomycin for 48 h. Non-transfected A549 cells that were infected with CCHFV at an MOI of 0.1 were used as positive cell controls. Cell morphology was monitored and 200 µL cell supernatant was harvested before nucleic acid extraction.

Virus replication decrease was assessed by determining the number of genome copies in 200 µL cell supernatant by RT-ddPCR.

### 4.6. Viral RNA Extraction

Template viral RNA from transfected cells and control cells were extracted from 200 µL culture supernatant using a DNA/RNA extraction kit (Geneaid, Taipei, Taiwan), according to the manufacturer’s protocol. The nucleic acid extraction was performed in the BSL-4 suite laboratory. The RNA elution was done in a volume of 50 µL of elution buffer and was stored at −80 °C until further use.

### 4.7. RT Droplet Digital PCR and Data Analysis

To investigate the inhibitory effect of all designed siRNAs in different concentrations (ranging from 10 to 50 nM), the RT-ddPCR assay was performed.

QX200 Droplet Digital PCR system (Bio-Rad, Hercules, CA, USA) was used to determine CCHFV copy number decrease triggered by siRNAs from supernatants. One-Step RT-ddPCR advanced kit for probes (Bio-Rad, Hercules, CA, USA) was used in our experiments. The RT-ddPCR reaction mixture consisted of 5 µL of a ddPCR Supermix, 2 µL reverse transcriptase, 1 µL 300 mM DTT, 900 nM CCHFV specific primers and 250 nM probe, 1 µL of sample nucleic acid solution, and nuclease-free H_2_O in a final volume of 22 µL. CCHFV specific primers and probes were based on Atkinson et al. publication [31] (Table 3). The entire reaction mixture was loaded into a disposable plastic cartridge (Bio-Rad) together with 70 µL of droplet generation oil for probes (Bio-Rad) and placed in the QX200 Droplet Generator (Bio-Rad). After processing, the droplets generated from each sample were transferred to a 96-well PCR plate (Bio-Rad) and heat-sealed with PX1^TM^ PCR Plate Sealer (Bio-Rad). PCR amplification was carried out on a C1000 Touch^TM^ Thermal Cycler with 96-Deep Well Reaction Module (Bio-Rad) using a thermal profile of beginning at reverse transcription: 50 °C for 1 h and 95 °C for 10 min, followed by 40 cycles of 95 °C for 30 s and 55 °C for 60 s, 1 cycle of 98 °C for 10 min, and ending at 4 °C. After amplification, the plate was loaded on the QX200 Droplet Reader (Bio-Rad) and the droplets from each well of the plate were read automatically. Positive droplets, containing amplification products, were partitioned from negative droplets by applying a fluorescence amplitude threshold in QuantaSoft^TM^ analysis software (Bio-Rad). The threshold line was set manually at 3780 amplitudes for every sample. Quantification of the target molecule was presented as the number of copies per µL of the PCR mix. All siRNAs in different concentrations were tested in three biological replicates.

### 4.8. Statistical Analysis

All experiments were repeated in three biological replicates. In our study, we compared the antiviral effect of selected effective siRNAs in different concentrations to the positive control to detect significant variations using the Student’s *t*-test. We compared the siRNAs inhibitory effect to the positive control because using the ON-TARGET plus non-targeting siRNA pool did not change the comparison result either. The measured dataset was statistically analyzed in the R environment [32]. The bar plots were created with ggplot2 R package [33]. During RT-ddPCR reactions, three biological replicates of siRNA inhibited CCHFV samples were used and we did not use technical replicates in the case of these siRNAs inhibited CCHFV samples since the three biological replicates include the technical replicate. However, the controls were used in three biological and three technical repeats. 

## Figures and Tables

**Table 1:** Calculated CC50 values by GraphPadPrism software during cell viability assay.

	siS1	siS2	siS6	siM1	siM6	siM17	siL1	siL3	siL4
**CC50 (nM)**	177.7	246.7	106.5	99.84	316.8	298.8	109.7	80.92	54.29
**R squared**	0.9758	0.9921	0.9376	0.8961	0.9842	0.8835	0.9334	0.9782	0.9038

**Table 2:** List of designed siRNAs.

siRNA ID	Position	Sequence			
siS1		S 5′:		GCGGCAACGAUAUCUUUGA	UU
	1651–1673	*mRNA:*	*GT*	*GCGGCAACGATATCTTTGA*	*GA*
		AS 3′:	UU	CGCCGUUGCUAUAGAAACU	
siS2		S 5′:		CCACAGUGUUCUCUUGAGU	UU
	26–48	*mRNA:*	*GC*	*CCACAGTGTTCTCTTGAGT*	*GT*
		AS 3′:	UU	GGUGUCACAAGAGAACUCA	
siS6		S 5′:		CAUGGACAUUGUAGCCUCU	UU
	1388–1410	*mRNA:*	*GA*	*CATGGACATTGTAGCCTCT*	*GA*
		AS 3′:	UU	GUACCUGUAACAUCGGAGA	
siM1		S 5′:		GGGCUUCCUUUCAAUAGAU	UU
	1134–1156	*mRNA:*	*AA*	*GGGCTTCCTTTCAATAGAT*	*TC*
		AS 3′:	UU	CCCGAAGGAAAGUUAUCUA	
siM6		S 5′:		GUCCAUACGAAGCUCUUGU	UU
	3173–3195	*mRNA:*	*TT*	*GTCCATACGAAGCTCTTGT*	*GC*
		AS 3′:	UU	CAGGUAUGCUUCGAGAACA	
siM17		S 5′:		CACGUCAGUACGUAAGUGU	UU
	19–41	*mRNA:*	*GG*	*CACGTCAGTACGTAAGTGT*	*CA*
		AS 3′:	UU	GUGCAGUCAUGCAUUCACA	
siL1		S 5′:		CAGGCCUUGAAGUCUUUAA	UU
	5264–5286	*mRNA:*	*GT*	*CAGGCCTTGAAGTCTTTAA*	*TG*
		AS 3′:	UU	GUCCGGAACUUCAGAAAUU	
siL3		S 5′:		GCCUCUUGAUAGGCACAAU	UU
	8442–8464	*mRNA:*	*GG*	*GCCTCTTGATAGGCACAAT*	*GT*
		AS 3′:	UU	CGGAGAACUAUCCGUGUUA	
siL4		S 5′:		GCCCUAUUUAGGGACAACU	UU
	10,080–10,102	*mRNA:*	*AA*	*GCCCTATTTAGGGACAACT*	*TG*
		AS 3′:	UU	CGGGAUAAAUCCCUGUUGA	

**Table 3:** Primers and probe information for the CCHFV RT dd-PCR assay based on Atkinson et al.’s publication.

Primer/Probe	Sequence (5′–3′)	Nucleotide Position
CCHF S1	TCTCAAAGAAACACGTGCC	1–19
CCHF S122	CCTTTTTGAACTCTTCAAACC	102–122
CCHF probe	(FAM) ACTCAAGGKAACACTGTGGGCGTAAG (BHQ1)	21–46
